# Movement speed is biased by prior experience

**DOI:** 10.1152/jn.00522.2013

**Published:** 2013-10-16

**Authors:** Ulrike Hammerbeck, Nada Yousif, Richard Greenwood, John C. Rothwell, Jörn Diedrichsen

**Affiliations:** ^1^Institute of Neurology, University College London, London, United Kingdom;; ^2^Institute of Cognitive Neuroscience, University College London, London, United Kingdom;; ^3^Division of Brain Sciences, Imperial College London, London, United Kingdom; and; ^4^National Hospital for Neurology and Neurosurgery, Queen Square, London, United Kingdom

**Keywords:** human, motor learning, movement speed, reaching

## Abstract

How does the motor system choose the speed for any given movement? Many current models assume a process that finds the optimal balance between the costs of moving fast and the rewards of achieving the goal. Here, we show that such models also need to take into account a prior representation of preferred movement speed, which can be changed by prolonged practice. In a time-constrained reaching task, human participants made 25-cm reaching movements within 300, 500, 700, or 900 ms. They were then trained for 3 days to execute the movement at either the slowest (900-ms) or fastest (300-ms) speed. When retested on the 4th day, movements executed under all four time constraints were biased toward the speed of the trained movement. In addition, trial-to-trial variation in speed of the trained movement was significantly reduced. These findings are indicative of a use-dependent mechanism that biases the selection of speed. Reduced speed variability was also associated with reduced errors in movement amplitude for the fast training group, which generalized nearly fully to a new movement direction. In contrast, changes in perpendicular error were specific to the trained direction. In sum, our results suggest the existence of a relatively stable but modifiable prior of preferred movement speed that influences the choice of movement speed under a range of task constraints.

individuals tend to move at a preferred speed: some talk slowly, whereas others walk fast (Slijper et al. 2009). Theoretical models suggest that the chosen speed is a compromise regarding the importance or reward value of the goal state (Xu-Wilson et al. 2009), the effort or energy needed to execute the movement (Mazzoni et al. 2007), and the cost of the wait before the goal is reached (Haith et al. 2012; Shadmehr 2010; Shadmehr et al. 2010; Tanaka et al. 2006). The motor system chooses a speed that optimizes a cost function (Todorov and Jordan 2002) representing a combination of these factors. For example, speed of walking with an arthritic hip could be a compromise between reaching the goal of catching the bus and minimizing pain.

Here, we ask whether the choice of movement speed, rather than being an optimal solution to an internal cost function, is also determined by the habit of moving at a specific movement speed that has formed during prior repetitive training. Previous studies have shown that a use-dependent learning mechanism strongly influences spatial characteristics (Diedrichsen et al. 2010; Verstynen and Sabes 2011). When Verstynen and Sabes (2011) trained people to move repeatedly in one direction, the variability of reaches in the trained direction decreased substantially. However, this came at the cost of biasing movements in less-frequently cued directions toward the trained direction. Therefore, movement speed may also be habitual in that a certain speed may be chosen because recent movements have tuned the system to this trained speed. If this habitual preference can be imposed by previous training, this would have important implications for ongoing training and treatment programs.

Our hypothesis was that training movements at a specific speed could similarly bias the speed of subsequent movements. Over 3 consecutive days of practice, a group of healthy young volunteers practiced a center-out arm-reaching task; half of them were trained to move fast, whereas the others were trained to move slowly. Before and after the training sessions, participants were required to make reaching movements within four different time windows. One of these was the same as the trained task; the three others were different. At the end of training, people in both training groups made more accurate movements at less variable speeds, but this came at the cost of biasing the speed in the untrained movements. Those who had been trained to move slowly tended to move slower than before training, whereas those who had been trained to move fast did the opposite. The changes in speed preference generalized to other movement directions, indicating that the underlying adjustment is relatively global.

## MATERIALS AND METHODS

### 

#### Subjects.

Eighteen healthy adults (mean age: 30.4 ± 8.30 SD yr, 7 males) without a history of upper-limb neurological or musculoskeletal disorder attended for 5 consecutive days. Experimental and consent procedures were approved by the University College London ethics committee.

#### Apparatus and stimuli.

**Fig. 1.**
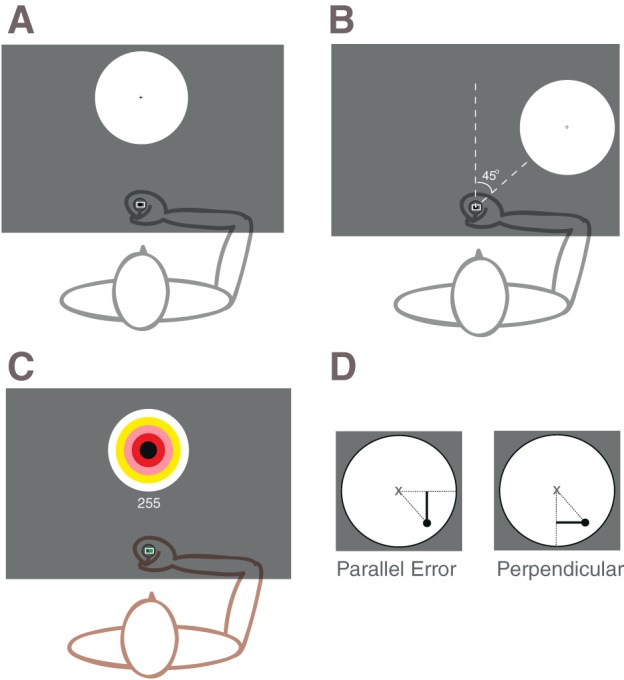
*A*: experimental display during pre- and posttesting. *B*: testing of transfer of performance to a target rotated 45° clockwise. *C*: diagram of experiment with target placed 25 cm directly in front of participants and bull's-eye scoring system. *D*: determining parallel and perpendicular error.

Participants were seated with their forehead supported on a headrest. Their semipronated right hand gripped a manipulandum underneath a horizontally suspended mirror. The mirror prevented direct vision of the hand and arm but showed a reflection of a computer monitor mounted above that appeared to be in the same plane as the hand. The visual display (Fig. 1*A*) comprised a 1-cm diameter starting box, a cursor (0.5-cm diameter) representing the position of the manipulandum, and a circular 10-cm diameter target with a small, black cross at its center, which was located 25 cm from the start box at an angle of either 0 or 45° (Fig. 1, *A* and *B*). At the start of a trial, a motor moved the participant's arm, and thereby the cursor, into the start box presented in midline. The glenohumeral joint was in ∼30° of elevation through flexion and 45° of abduction, and the elbow was at ∼90° flexion. The duration of each trial was identical irrespective of the allowed movement time (i.e., 2,320 ms), but reaction time was not enforced and was not included in the trial time. The starting signal, a change from the target (Fig. 1*A*) from an outline to a solid color, appeared 100 ms after trial initiation. Participants were instructed to start moving in their own time after the starting signal. At movement onset, the cursor disappeared and reappeared, showing the hand position, when the allowed movement time had passed. Reappearance of the cursor indicated the end of the trial and provided feedback of reach endpoint accuracy. The feedback was displayed for 300 ms, after which the robotic manipulandum moved the arm back to the starting position. The hand was held in the starting position until the end of the trial time. Shorter movement times therefore had longer wait times at the end of a trial for initiation of the next trial. Participants familiarized themselves with the task by performing 10 reaches with continual visual feedback at 900 and 300 ms as well as 10 reaches without visual feedback at 300 ms.

#### Pre- and posttest.

On *days 1* and *5* (pre- and posttraining), reaching accuracy was established at four different movement times, 300, 500, 700, and 900 ms, by performing blocks of 45 trials presented in a random order. We performed the reaches at comfortable reaching speeds (500 and 700 ms; Soechting et al. 1995) as well as very fast (300-ms) movement times and slow movements (900 ms; Nishikawa et al. 1999). The 1st 5 trials of each block were used for practice, and the subsequent 40 trials were analyzed. All 4 movement times were performed with targets at 0 or 45° (Fig. 1, *A* and *B*), and the order of conditions was randomized. In all cases, the aim was to end the movement as close as possible to the cross in the center of the target.

Participants were instructed to perform the reach within a predetermined movement time. The movement time was enforced by “dropping” the cursor at the hand position that was achieved when the allowed movement time had passed, whether the hand had stopped moving or not. This cursor position was used for determining endpoint error. Any further corrective movement after this time did not influence the measurement. This arrangement means that movement times cannot be longer than the allowed time, although shorter movement times are possible.

#### Training.

On the 2nd day, participants were randomly assigned to either a fast (cursor reappears after 300 ms) or a slow (cursor reappears after 900 ms) training group and, during *days 2*, *3*, and *4*, trained to reach accurately at these movement times for 630 reaches per day (7 blocks of 90 repeats). Each training session lasted ∼1 h. During training, the target size and location at 0° remained unchanged but was now displayed as a bull's eye (Fig. 1*C*, training display) with concentric colored circles at 1-, 2-, 3-, 4-, and 5-cm radius. This provided feedback of results during training to maintain interest and incentivize participants. Points were awarded after every reach (5 points maximum when absolute error < 1 cm from the center of the target, 4 points for <2-cm, 3 points for <3-cm, 2 points for <4-cm, and 1 point for <5-cm error) and accumulated throughout the block with a maximum score of 450 points per block. Participants were encouraged to increase their points per block and were reminded of their performance on the previous block and the previous day(s). Additionally, participants were informed that a monetary prize would be awarded to the participant demonstrating the greatest improvements due to training.

#### Data analysis.

Our main measures were the change in maximum tangential movement speed (centimeters per second) induced by training at different movement speeds and change in endpoint error, defined as the vector between the target center and the position of cursor presentation at the end of allocated movement time. The error on each trial was divided into a component along the movement direction (parallel error) and one orthogonal to the movement direction (perpendicular error; Fig. 1*D*). Thus the mean squared Euclidean error is equal to sum(perpendicular error^2^)/*n* + sum(parallel error^2^)/*n*.

All illustrations and statistical testing use the root mean squared error for each component. A two-factorial (2 × GROUP, 4 × MOVEMENT TIME) repeated-measures ANOVA (rmANOVA) was used with Greenhouse-Geisser correction for nonsphericity as indicated.

Correlation was performed on a subject-by-subject basis and is expressed as the *r* value as well as the level of significance.

## RESULTS

### 

#### Speed choice during pretest.

**Fig. 2.**
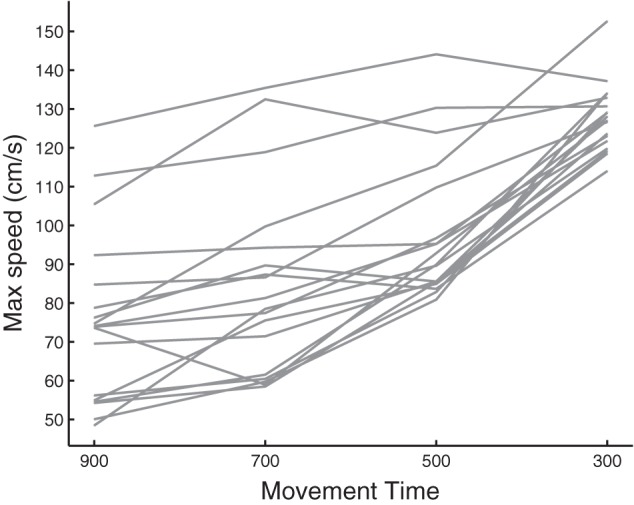
Illustration of each participant's preferred movement speed at the 4 set movement times (in milliseconds).

The constraint on movement speed we imposed for the four different target speeds was asymmetric as can be observed (Fig. 2) by the limited spread of data at the fastest speed and much greater variance at low target speeds. The choice of target speed differed, however, between subjects, and this choice was relatively stable, demonstrated by the subject-by-subject correlation between the slowest target speed and the other slow target-speed conditions [900–700, *r*_(16)_ = 0.894, *P* < 0.001; 900–500, *r*_(16)_ = 0.868, *P* < 0.001]. Because the constraint of moving very fast in the 300-ms condition prevents a true choice of speed, we observe that the effect of the prior is abolished [900–300, *r*_(16)_ = 0.261, *P* = 0.295].

#### Movement changes during training.

**Fig. 3.**
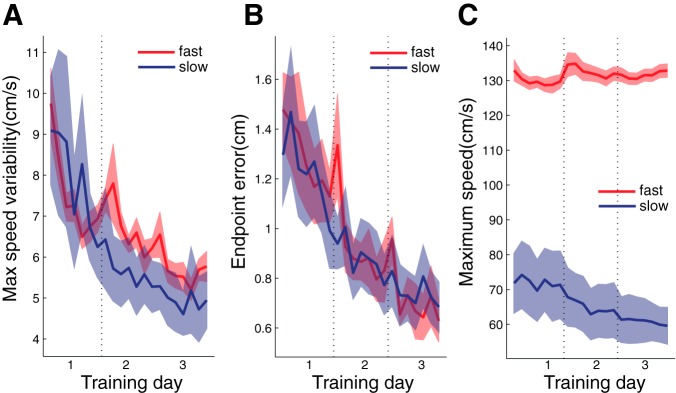
Reaching performance during training days (*days 2*–*4*). *A*–*C* show how performance in the training blocks (9/day) changes over the 3 days in the fast (red) and slow (blue) training group. *A*: variability (SD) of peak movement speed. *B*: root mean square endpoint error. *C*: mean peak movement speed.

Over the 3 days of training, participants reduced the variability of peak movement speed (Fig. 3*A*). A 2-way rmANOVA on the SD of the maximal speed with the factors GROUP (fast vs. slow) and TIME (1st vs. 3rd training day) showed a significant effect of TIME [*F*_(1,16)_ = 53.451, *P* < 0.001]. Simultaneously, participants also showed a significant improvement of their movement error [Fig. 3*B*; *F*_(1,16)_ = 34.708, *P* < 0.001], which was similar in the slow and fast training group.

For the fast group, the average movement speed remained relatively constant over the 3 days (Fig. 3*C*), whereas it decreased slightly in the slow group, resulting in a significant TIME × GROUP interaction [*F*_(1,16)_ = 5.762, *P* = 0.029] and a marginally significant effect of TIME [*F*_(1,8)_ = 5.021, *P* = 0.055]. This is probably because the slow group moved at their preferred speed at the beginning of training and waited at the end of the movement for the reappearance of the cursor. Over the training days, they abandoned this “move and wait” strategy and slowed down their movements. This strategy was not available to the fast group, who were moving at close to their maximum speed anyway.

The interval between the onset of each movement was matched between conditions. Since the percentage of correct movements did not differ, the average rate of reward during the training phase was the same in each group (Table 1).

**Table 1.** Statistical summary comparing reward rate during training between the fast and slow training group

	Mean (SE)		
Reward Rate	Fast	Slow	Significance
Percentage correct	93.4% (0.68)	93.32% (1.10)	*t*(16) = 0.075, *P* = 0.941
Trial duration	2,663.9 ms (19.7)	2,747.8 ms (55.5)	*t*(16) = −1.426, *P* = 0.173
Reward rate	1.75/s (0.02)	1.70/s (0.05)	*t*(16) = 1.010, *P* = 0.328

Detailed comparison of reward rate between groups during the training phase. The percentage of correct movements was very similar. Trial duration could be altered by the reaction time, but the groups did not differ. Therefore, reward rate (points per second) between the groups was the same.

#### Influence of training on movement speed.

**Fig. 4.**
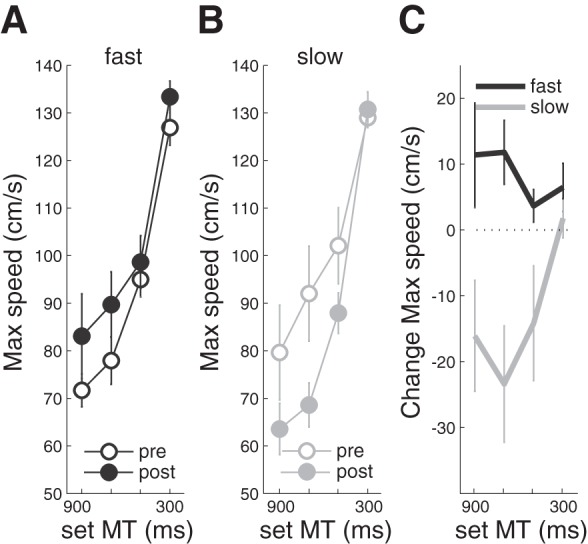
Comparison of changes in maximum movement speed (*A*–*C*) due to training. *A*: data from the fast training group before (pre; open) and after (post; closed) training for movement times (MT) of 900, 700, 500, and 300 ms. *B*: slow training group measures. *C* plots prepost change scores of maximum speed.

Before and after the 3 days of training, performance was tested at four different speeds by changing the time at which the cursor reappeared (300, 500, 700, and 900 ms). Figure 4, *A* and *B*, shows that participants adapted peak velocity to match the time available for moving.

By the end of training, participants had developed a bias toward the trained speed. The fast group (Fig. 4*A*) moved quicker at all speeds except the highest movement speed where faster movements were difficult. The slow group (Fig. 4*B*) moved more slowly at all speeds except the highest movement speed where lower movement speeds would lead to task failure. This was confirmed in a two-way rmANOVA on the change in movement speed (Fig. 4*C*) from pre- to posttest. There was a significant main effect of TRAINING GROUP [slow vs. fast; *F*_(1,16)_ = 9.597, *P* < 0.007] as well as a significant GROUP × TARGET SPEED interaction [*F*_(1.932,30.91)_ = 3.415, *P* = 0.047]. This interaction was not driven by the significant changes that are seen at the trained movement speed as a 2 × 2 rmANOVA for the untrained movement speed (500 and 700 ms) also yielded a significant GROUP × TARGET SPEED interaction [*F*_(1,16)_ = 11,172, *P* = 0.004] as well as a significant effect of GROUP [*F*_(1,16)_ = 1.436, *P* = 0.011].

**Fig. 5.**
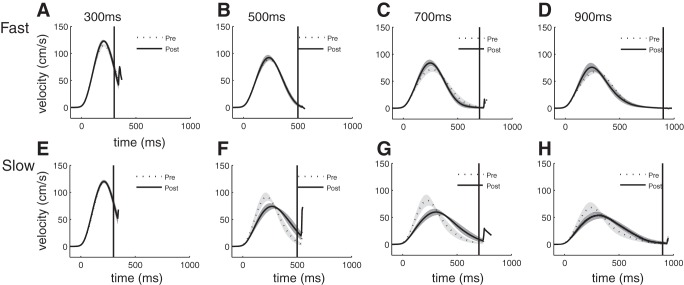
Changes to velocity profiles induced by training. Group average (±SD of individuals) of fast (*A*–*D*) and slow (*E*–*H*) groups are at 300 ms (*A* and *E*), 500 ms (*B* and *F*), 700 ms (*C* and *G*), and 900 ms (*D* and *H*).

Training did not only alter peak velocity, but also the velocity profile of the movement (Fig. 5). The profiles for the slowest movement times were highly skewed before training, demonstrating that participants moved and then waited for the end of the movement time at the target. Training at the slow speed led to more symmetric velocity profiles by delaying the time of peak speed (Fig. 5, *E*–*H*). This was not the case for the group that trained at the fast speed (Fig. 5, *A*–*D*). The time at which peak speed was reached exhibited a significant GROUP × TARGET SPEED interaction [*F*_(3,24)_ = 7.460, *P* = 0.001] as well as a significant effect of GROUP [*F*_(1,16)_ = 8.026, *P* = 0.012].

**Fig. 6.**
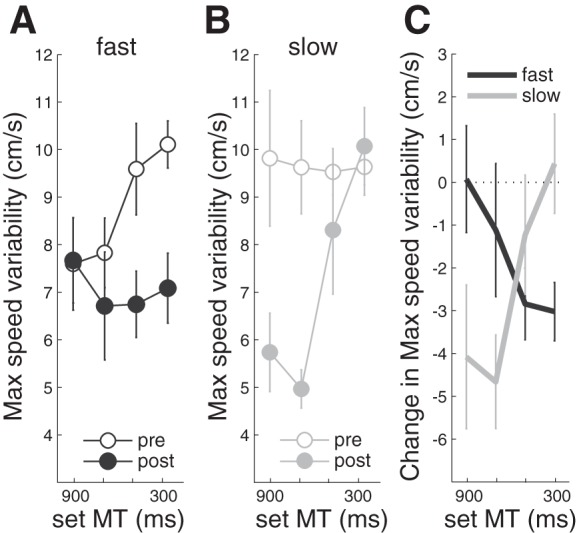
Comparison of changes in maximum speed variability (*A*–*C*) due to training. *A*: data from the fast training group before (open) and after (closed) training for movement times of 900, 700, 500, and 300 ms. *B*: slow training group measures. *C* plots prepost change scores of maximum speed variability.

Reduced variability of peak speed observed during training (Fig. 3*B*) was also evident when inspecting the pre- and posttraining performance. Interestingly, the reduction was specific to the trained speed. The fast training group reduced the variability of movement speed mostly for the higher speeds (Fig. 6*A*), whereas the slow group decreased it mostly for the lower speeds (Fig. 6*B*). This training-specific effect can be seen most clearly in the pre- to posttest difference plots (Fig. 6*C*). A two-factor rmANOVA confirmed that there was a highly significant GROUP × TARGET SPEED interaction [*F*_(3,48)_ = 5.047, *P* = 0.004]. Between-group, post hoc *t*-tests on the reduction of movement-speed variability was significantly changed for the fast training group [*t*_(16)_ = −2.570, *P* = 0.021] and showed a trend for the slow group [*t*_(16)_ = −2.037, *P* = 0.059]. The combination of a training-dependent movement-speed bias and a specific reduction in speed variability suggests similar mechanisms as has been observed for training-induced changes in movement direction (Verstynen and Sabes 2011).

#### Changes in movement accuracy.

**Fig. 7.**
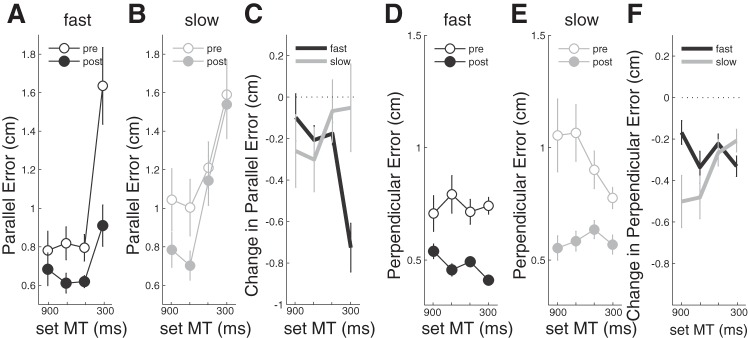
Measures of parallel error (*A*–*C*) and perpendicular error (*D*–*F*) at each of the 4 movement times before (open) and after (closed) training. *A* and *B*: parallel error for the fast and slow training groups, respectively. *D* and *E*: perpendicular error for the fast and slow training groups. *C* and *F*: prepost difference scores in parallel and perpendicular error.

Movement accuracy improved with training. When separated into parallel and perpendicular components, we found that parallel error (Fig. 7, *A*–*C*) decreased, particularly for the fastest movements in the fast group (Fig. 7*A*). An rmANOVA on the prepost differences (Fig. 7*C*) confirmed a significant TRAINING GROUP × TARGET SPEED interaction [*F*_(3,48)_ = 9.164, *P* < 0.001] with a post hoc *t*-test showing that the improvement was significantly greater in the fast than the slow group for the fastest target speeds [*t*_(16)_ = −2.739, *P* = 0.013]. For the shortest movement duration, the parallel error correlated strongly with the movement speed on a trial-by-trial basis such that slow movement undershot and fast movement overshot the target [*r* = 0.795 ± 0.061 (SE)]. Therefore, reductions in speed variability would automatically lead to reduced parallel error. Indeed, on a subject-by-subject level, reductions in the variability of the parallel error were significantly correlated with the reduction in peak speed variability [*r*_(16)_ = 0.649, *P* = 0.004].

Perpendicular error was also reduced after training in both groups (Fig. 7, *D* and *E*), demonstrated by a significant effect of TIME in two-way rmANOVAs on the prepost data [*F*_(1,16)_ = 58.099, *P* < 0.001]. The improvement was particularly evident in the slow training group when moving at the longest movement times [*t*_(8)_ = −2.836, *P* = 0.022]. The latter was confirmed by a two-way rmANOVA on the prepost training change (Fig. 7*F*) showing a TRAINING GROUP × TARGET SPEED interaction [*F*_(1.798,28.762)_ = 4.158, *P* = 0.030].

#### Generalization to another movement direction.

**Fig. 8.**
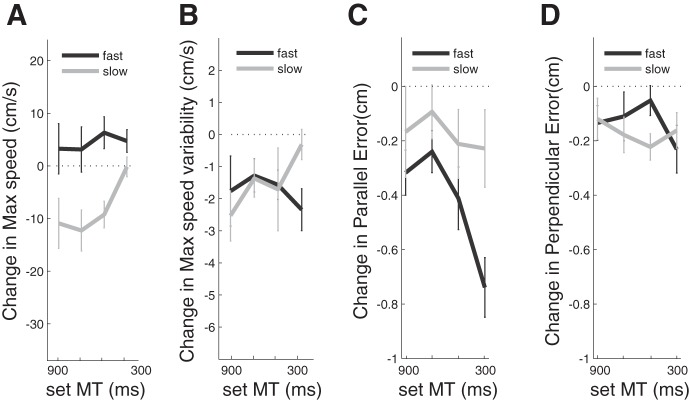
Transfer to target positioned 45° clockwise. *A*: change (pre/post) in maximum movement speed in the fast and slow training groups. *B*: change in endpoint error. *C*: perpendicular error. *D*: parallel error.

Finally, we assessed how training changed preferred speed and endpoint accuracy for movements aimed at a target that was 45° clockwise to the trained direction (Fig. 1*B*). Figure 8*A* shows that, compared with the baseline (pretraining) data, movements at all target speeds were slower in the slow training group and faster in the fast training group with a significant effect of TRAINING GROUP [*F*_(1,16)_ = 11.391, *P* = 0.004]. The size of the effect was, however, smaller than for movements made in the trained direction (compare with Fig. 4*C*). Peak speed variability was also reduced in the new direction [significant effect of TIME, *F*_(1,16)_ = 8.664, *P* = 0.010] but to a lesser degree than in the trained direction (Fig. 8*B*).

Analysis of the parallel component of the error (Fig. 8*C*) demonstrated an effect of GROUP [*F*_(1,16)_ = 4.227, *P* = 0.056]. The fast group significantly improved their parallel error at the fast movement speed [post hoc *t*-test: *t*_(16)_ = −2.492, *P* = 0.024]. In contrast, the perpendicular error (Fig. 8*D*) showed only small, nonsignificant improvement for both groups, suggesting that the acquired improvement in perpendicular accuracy was specific to the trained movement direction.

## DISCUSSION

The present results suggest that the speed with which people move is not solely determined by an optimality criterion that combines task constraints, the reward value of the target, and intrinsic costs of movement (Todorov and Jordan 2002). Instead, the choice of movement speed is partly habitual, depending on prior experience and modifiable through prolonged training at a specific speed. The experiments used a standard center-out reaching task in which movement speed was asymmetrically constrained: the cursor indicating hand position was removed at the start of movement and was redisplayed after 300–900 ms as a stationary cursor that indicated the end point. If the cursor appeared, for example after 500 ms, then movements that were too slow and had not reached the target by that time would be penalized for undershooting. However, maximum speed was not specified. Fast movements arrive early at the end point, and the only consequence is that participants have to wait a short time before the cursor reappears. Thus this method effectively enforces a minimum movement speed while leaving participants free to choose as fast a speed as they are comfortable with. This redundancy allowed us to measure the influence of the preferred (or habitual) movement speed. Although we could have also used a task in which we simply asked people to move at their preferred speed in the absence of any constraints, such experiments can be highly susceptible to influences of task instructions. We therefore used this partly constrained version, which also allowed us to test the influence of an acquired habitual speed across different speed constraints.

After training, participants were tested at movement speeds that had not been trained. We observed that their preferred speed was altered: the fast training group moved faster, and the slow training group slower. This change was also evident in the velocity profiles demonstrating a change in maximum speed as well as an alteration of the time of this speed (Flash and Hogan 1985). Simultaneously, each group showed a marked reduction in the trial-by-trial variability of peak speed when tested at their trained movement speed. We suggest that this effect is a consequence of use-dependent learning in which the movement speed that enhances success during training biases the speed of subsequent movements. In essence, our experiment constitutes a temporal version of the spatial phenomenon described by Verstynen and Sabes (2011), who found that moving repeatedly to a single target reduced the directional variability to the trained target but also biased the direction of movements to nearby targets. In the experiments of Verstynen and Sabes (2011), the spatial effects occurred rather rapidly within a single experimental session. In the present case, since we were interested in lasting long-term consequences of training, we did not examine how the effect changed during the training days. Nevertheless, the effects were consolidated and still present 1 day after the final training session.

The bias toward the practiced speed of movement transferred to a different movement direction. Although we did not assess how far this changed preference would generalize (i.e., to different movement with the same effector, movements with the other hand, or with other body parts), our results suggest that habitual movement speed is influenced by training on a more global level than visuomotor adaptation (Krakauer et al. 2006) or changes in proprioceptive accuracy (Ostry et al. 2010).

An unresolved question concerns the exact nature of the change in preferred movement speed. We have here favored the idea that training generates an attractor that biases movements toward the speed at which the training was performed in the same way as repeatedly practicing movements in one direction biases subsequent movements toward the same direction (Verstynen and Sabes 2011). However, it is also possible that training at different speeds influences movement vigor in general (Haith et al. 2012). Because we trained participants only on the slowest and fastest condition of our tested range, our data on speed bias do not allow us to differentiate between these two explanations. However, an attractor-like mechanism can simultaneously account for the specific reduction in the movement-speed variability, as it would predict that movement speeds nearer to the trained velocity would be pulled toward the learned prior. It is unclear how a general change in movement vigor would account for this result.

Another important question is whether the change in the preferred movement speed was induced solely by the act of moving at this speed (Diedrichsen et al. 2010; Verstynen and Sabes 2011) or whether the experience of successful movements at that particular speed was critical (Huang et al. 2011). Further experiments are required to tease apart the role of these two factors.

Nonetheless, our finding provides a new and important insight into how the motor system determines movement speed for any given task. All previous models share the common assumption that the chosen speed is a moment-by-moment compromise between external and internal constraints. For example, in the model by Tanaka et al. (2006), movement duration is set as low as possible while still fulfilling the accuracy constraints: faster movements would entail more signal-dependent noise and reduce accuracy. An external factor that speeds up movement times is the reward value of the goal. Experiments in the macaque show that eye movement speed can be >20% higher when the target is rewarded compared with nonrewarded (Bendiksby and Platt 2006). Similar results have been described in humans (Shadmehr et al. 2010). Most recent models combine these factors by proposing that the motor system strives to maximize the overall rate of reward, which leads to a compromise between the probability of success and a hyperbolic discounted reward value (Haith et al. 2012; Shadmehr 2010). These types of models cannot account for our results, as the reward probability (i.e., success rate) as well as the rate of reward (Haith et al. 2012) were matched across the two training groups.

Our experiments demonstrate that the speed we move at is also influenced by past experience. They indicate that the motor system does not approach each task as a blank slate and reoptimize the preferred movement speed de novo but rather carries with it preferences for certain speeds of movement. The existence of such a preference, even at the start of training, could be observed in our slow training group, which moved faster than necessary and therefore had to wait at the goal. Our results also demonstrate that this natural preferred movement speed can be modified through repeated practice at an enforced speed.

This insight may also have important implications for understanding and treating clinical movement disorders. Slowness of movement is a common feature but is also observed in healthy aging (Gill et al. 1997; Mazzoni et al. 2007). These changes are often explained as an optimal response to a changed speed-accuracy trade-off, i.e., the movement may be slowed down to be able to achieve the necessary level of spatial accuracy required by everyday task. Alternatively, slowness of movement may be consequence of a changed reward-to-effort or -cost ratio (Mazzoni et al. 2007). Our findings suggest that, although the initial slowing may be indeed caused by a combination of these factors, the slowness may be further consolidated by the habitual formation of a behavioral bias that slows down all movement. Thus, even if the underlying deficit was removed, slowness of movement may persist due to a persistent change in the preferred movement speed. Following this idea, it may not be enough to improve accuracy of movement through physical therapy; it may also be necessary to overcome the habitual slow movement speed by training at speeds that are higher than the preferred set point.

We also found practice-related improvements in movement accuracy. Part of this was linked to reduced variability of maximal movement speed: this was apparent especially for the parallel error (parallel to the target direction) for the fast training group, in which the reduced error almost fully accounted for the reduced variability in peak velocity. Gains in perpendicular accuracy were more specific to the trained movement direction. This indicates that the accuracy gains may depend on learning the muscle and joint dynamics for a specific movement direction, leading to relatively narrow generalization (Orban de Xivry et al. 2011).

Our experiment demonstrates that chosen movement speed, even under relatively constrained conditions, is influenced by prior training. The effects were visible even 1 day after the last training day and generalized to a different movement direction. Although these findings imply general and long-lasting changes to preferred speed, we have not yet established whether these effects generalize outside of the experimental setting and over what time period they disappear after the end of training. Nonetheless, our findings demonstrate that the motor system reoptimizes the movement speed for any given task by taking into account a strong internal prior that is shaped by recent experience.

## GRANTS

This work was supported by grants from the Stroke Association (TSA 2010/06) to J. C. Rothwell and U. Hammerbeck, the Chartered Society of Physiotherapy Charitable Trust (Grant PRF 10/09) to U. Hammerbeck, and University College London-Biomedical Research Council support to R. Greenwood.

## DISCLOSURES

No conflicts of interest, financial or otherwise, are declared by the author(s).

## AUTHOR CONTRIBUTIONS

U.H., N.Y., R.G., J.C.R., and J.D. conception and design of research; U.H. performed experiments; U.H., N.Y., J.C.R., and J.D. analyzed data; U.H., R.G., J.C.R., and J.D. interpreted results of experiments; U.H. prepared figures; U.H. drafted manuscript; U.H., N.Y., R.G., J.C.R., and J.D. edited and revised manuscript; U.H., N.Y., R.G., J.C.R., and J.D. approved final version of manuscript.
